# Airborne Microbiome of Tropical Ostrich Farms: Diversity, Antibiotic Resistance, and Biogeochemical Cycling Potential

**DOI:** 10.3390/ani16060880

**Published:** 2026-03-12

**Authors:** Yu Yang, Junchi Wang, Zetong Wang, Cheng Li, Xiaolei Hu, Songdi Liao, Lizhi Wang

**Affiliations:** Key Laboratory of Agro-Forestry Environmental Processes and Ecological Regulation of Hainan Province, School of Environmental Science and Engineering, Hainan University, Haikou 570228, China; yangyu_mail@163.com (Y.Y.); wangjunchi987@163.com (J.W.); 24210830000017@hainanu.edu.cn (Z.W.); 996173@hainanu.edu.cn (X.H.); 996983@hainanu.edu.cn (S.L.); wanglz@hainanu.edu.cn (L.W.)

**Keywords:** bioaerosols, microbial diversity, ostrich, 16S rRNA sequencing, metagenomics, antibiotic resistance genes

## Abstract

The expansion of tropical specialty livestock farming raises urgent concerns about airborne pathogen and antibiotic resistance dissemination. Ostrich farming, characterized by high-density stocking and feed exposure, yet their microbial ecology remain poorly characterized. This study analyzed 48 bioaerosols samples from an ostrich farm in Hainan, China, across dry and rainy seasons using 16S rRNA sequencing and metagenomics. The bacterial community were dominated by Firmicutes, Proteobacteria, and Actinobacteria, followed by *Staphylococcus*, *Bacillus*, and *Acinetobacter* as predominant genera, with particle size significantly shaping their structure. Large particles (>7.0 μm) carried higher species richness, while medium particles (2.1–3.3 μm) exhibited the highest diversity and evenness. Notably, small particles (0.65–1.1 μm), which can penetrate deep into the lungs, were enriched with *Brevibacillus* and *Corynebacterium*. Metagenomic analysis identified 638 antibiotic resistance genes (ARGs), dominated by efflux pump-associated determinants. The detection of clinically relevant ARGs (e.g., *mcr-1* and *bla_TEM_*) reflects the genetic potential of the airborne resistome, rather than confirmed resistance phenotypes or active horizontal gene transfer. Functional analysis revealed a strong potential for organic matter degradation, driven by abundant carbohydrate-active enzymes (CAZymes) and their corresponding CAZyme genes, as well as a nitrogen cycle dominated by assimilation and reduction pathways, while genes for nitrogen fixation and nitrification were absent. Our findings demonstrate that ostrich farming enhanced airborne microbial diversity and functional potential, facilitating the ARG dissemination and nitrogen transformation. This study provides critical insights into the ecological and health risks of bioaerosols in tropical livestock farms, informing environmental monitoring and risk management strategies.

## Simple Summary

Ostrich farming is rapidly developing in tropical regions, but little attention has been given to the microorganisms and antibiotic resistance carried in farm air. In this study, we collected airborne bioaerosols from different seasons, functional areas, and particle size fractions of an ostrich farm in Hainan, China, and analyzed them using 16S rRNA sequencing and metagenomics. The airborne bacterial community was mainly composed of Bacillota, Proteobacteria, and Actinobacteriota, with genera such as *Staphylococcus*, *Bacillus*, and *Acinetobacter* being dominant. Seasonal changes, farming activities, and particle size characteristics jointly shaped the structure of the airborne microbiome. We detected a wide range of ARGs, including β-lactam resistance genes dominated by OXA-type determinants and fluoroquinolone resistance genes such as *gyrA*, *gyrB*, and *parC*. Functional genes indicated that airborne microorganisms may participate in carbon and nitrogen cycling, suggesting potential ecological importance. These findings show that bioaerosols in ostrich farms may act as a reservoir of antibiotic resistance and functionally active microbes, highlighting the need for enhanced environmental monitoring and One Health-based risk management.

## 1. Introduction

Antibiotic resistance has become a major global public health concern [1], with livestock and poultry farms recognized as important reservoirs and dissemination hubs of ARGs [2]. Although many countries, particularly developed ones, have restricted or banned the use of antibiotics for growth promotion, antibiotics are still used in animal production for the treatment of diseases. This has led to the continuous accumulation of ARGs in the gut microbiota of animals. These genes can be released into the environment through feces, wastewater, and bioaerosols, becoming a significant source of antibiotic resistance pollution in the environment [3]. Persistent residues in manure, dust, and aerosols exert continuous selection pressure, enriching ARGs even at sub-inhibitory concentrations via bacterial adaptation and horizontal gene transfer (HGT) [4]. ARG types and abundances vary across livestock systems: for example, Zhu et al. detected 13 ARG classes in layer hen feces and soil [5], while Yang et al. found 107 ARG classes in air sample [6], highlighting the critical role of airborne transmission in ARG dissemination. Tetracycline resistance genes are prevalent in pig and cattle farm air, and macrolide resistance genes (*ermA*, *ermB*) are common in poultry houses [7,8]. Different antibiotic classes vary in environmental stability: tetracyclines persist and adsorb to particulates, while β-lactams degrade more rapidly [9]. Airborne bioaerosols can be inhaled, and high microbial density in intensive farms promotes HGT between pathogenic and non-pathogenic bacteria [10]. Commensal and environmental bacteria serve as long-term ARG reservoirs, and elevated ammonia (NH_3_) and hydrogen sulfide (H_2_S) can further influence microbial communities and ARG distribution [11]. Despite studies in pigs, chickens, and cattle, ostrich farm bioaerosol ARGs and microbial composition remain largely unexplored.

Given that ARGs can be transported via airborne particles, studying bioaerosols in livestock farms is essential for assessing both environmental and public health risks. The expansion of modern animal husbandry intensifies these risks, as bioaerosols disperse pathogens and ARGs [12]. Previous studies have reported that microbial concentrations in bioaerosols from conventional livestock farms, such as pig and poultry operations, range from 10^3^ to 10^6^ colony-forming units (CFU) per cubic meter (m^3^) [13], which are substantially higher than those observed in environments such as sewage treatment plants and landfills [14,15,16]. These bioaerosols serve as a linkage between animal-associated microorganisms and the surrounding air environment, thereby creating potential exposure pathways relevant to health risks. Compared with conventional livestock systems, bioaerosols originating from specialty livestock farms have received far less attention. China currently recognizes 17 traditional and 16 specialty livestock species [17], among which ostrich farming has attracted increasing interest due to its rapid development and economic value. Such intensive farming may further enhance airborne microbial exposure, including ARG dissemination. However, limited investigation of bioaerosols from specialty livestock systems has constrained understanding of their environmental dissemination processes and related health concerns.

The ostrich originated in Africa and the Arabian Desert region and is the largest extant herbivorous bird species. Large-scale ostrich farming began in South Africa in the 19th century and has a history of more than 150 years. South Africa remains the global center of the ostrich industry, with an annual standing population of approximately 300,000 birds. Subsequently, ostrich farming gradually expanded to countries such as the United States, Australia, and Egypt [18]. The ostrich industry in China developed relatively late. In 1992, the Jiangmen Livestock Farm in Guangdong Province first introduced eight African ostriches for breeding, and nationwide promotion began in 1993. At present, China has established more than 400 ostrich farms with an estimated population of approximately 100,000 birds, indicating a developed industry foundation. With global population growth and increasing demand for high-quality animal protein [19,20], alternative livestock systems such as ostrich farming are gradually supplementing traditional animal husbandry, and incorporation into conventional poultry systems can help alleviate protein supply pressure [21]. In terms of production performance, ostriches grow rapidly, reaching 80–100 kg within the first year [22]. Adults typically weigh 120–150 kg, with a dressing percentage of 57–58% [23]. In addition, ostriches produce ~40 eggs per laying season, with a rate of 58.59%, enhancing their economic value. Moreover, the nutritional and economic value of ostrich products contributes to the expansion of intensive farming practices that may influence airborne microbial dynamics. From a product perspective, ostrich meat exhibits notable nutritional advantages, commonly summarized as the “three lows and five highs”: low fat, low cholesterol, low energy, and rich in iron, calcium, selenium, zinc, and high-quality protein. Fat content is as low as 3 g/100 g, protein 22–24 g/100 g [24,25,26]. Moreover, meat contains a high proportion of polyunsaturated fatty acids (27.54 ± 1.01), is rich in Omega-3, and has a relatively high mineral content [27]. Cholesterol is ~65.63 mg/100 g, 1.08% of total fat. Together with its tender, juicy texture and distinctive flavor, these attributes enhance its popularity among health-conscious consumers [26,28]. In addition to meat, ostrich by-products such as skin and eggs possess high added value, supporting a diversified utilization model with promising market prospects. However, high-density outdoor ostrich farming provides an ideal environment for harmful bioaerosols. The airborne microbiome—including composition, resistome, and functional potential—remains poorly characterized, hampering effective risk assessment.

Airborne microorganisms are not only critical contributors to environmental health risks but also play fundamental ecological roles in biogeochemical processes such as carbon and nitrogen cycling [29]. Beyond public health implications, understanding the ecological functions of airborne microbes is necessary, as air microbial communities participate in organic matter degradation, carbohydrate metabolism, and nitrogen transformation, thereby influencing material cycling and the ecological stability of livestock farming environments [30]. CAZymes are key functional proteins responsible for the synthesis, modification, and degradation of carbohydrates in microorganisms, typically accounting for 1–3% of microbial genomes [31]. The composition and abundance of CAZyme families reflect microbial metabolic potential in organic matter decomposition, carbohydrate utilization, and energy conversion [32]. Previous studies have revealed that Actinobacteria and Proteobacteria are the main sources of CAZymes, while certain specialized genera such as Asticcacaulis and Cytophaga exhibit substrate specificity in complex organic matter degradation, causing variations in CAZyme composition and abundance across environments [33]. External factors, such as fluctuations in temperature and humidity, animal and human activities, and changes in organic load, can shape microbial community structure and modulate carbohydrate metabolism. Environmental conditions, particularly temperature and moisture, strongly affect microbial composition and functional processes, including carbon metabolism, whereas variations in organic load are linked to changes in community structure and metabolic performance [34].

Nitrogen cycling, an essential biogeochemical process that maintains nutrient balance and ecosystem stability, includes multiple stages such as nitrogen fixation, nitrification, denitrification, assimilatory reduction, and dissimilatory reduction, jointly driven by diverse functional microorganisms [35]. Microbial diversity and community composition significantly influence nitrogen transformation rates and N_2_O emissions, with high diversity or rapid shifts promoting organic nitrogen conversion, whereas low-diversity communities often enhance denitrification efficiency [36]. Airborne microorganisms and their metabolites, together with organic dust, fecal particles, and feed residues, form a complex bioaerosol system. The distribution of CAZymes and nitrogen-cycle-related functional genes within this system reflects the ecological potential of microbial communities in organic matter degradation, carbon-energy metabolism, and nitrogen transformation. Qin et al. identified functional genes related to carbohydrate metabolism and denitrification in urban airborne microbial communities, suggesting that airborne microbes may play important roles in elemental cycling [37]. Nevertheless, the carbon and nitrogen cycling potential and ecological functions of airborne microbial communities in specialty livestock farms, particularly ostrich farms, remain largely unexplored, limiting a comprehensive understanding of bioaerosol ecological functions and environmental risks.

In view of the potential importance of airborne microbiota in farms for the dissemination of ARGs and for carbon and nitrogen cycling, this study investigated bioaerosols across different seasons and functional zones in a specialty ostrich farm in Hainan. By integrating 16S rRNA high-throughput sequencing with metagenomic analysis, we characterized airborne microbial community composition and diversity, ARG profiles, and functional genes involved in carbon and nitrogen cycling. This study aimed to elucidate microbial diversity patterns, identify ARG types and potential transmission pathways, and assess ecological functions related to carbohydrate degradation and nitrogen transformation in ostrich farm bioaerosols. By comparing these results with those from conventional livestock systems (e.g., swine, poultry, and cattle farms), this work provides differentiated insights into biosafety management in specialty farming environments. Overall, the findings establish a foundational dataset and broaden the understanding of air microbiology in non-traditional livestock systems, offering theoretical support for ecological management, antimicrobial resistance risk control, and the sustainable development of specialty livestock farming.

## 2. Materials and Methods

### 2.1. Selection and Description of the Breeding Site

**Figure 1:**
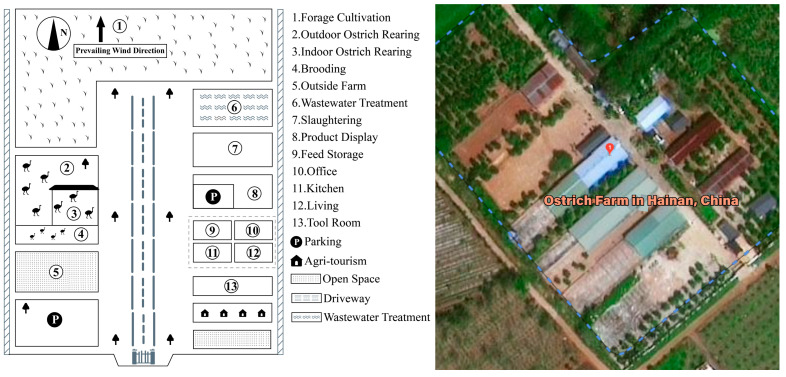
Layout of the ostrich farm and the distribution of sampling sites across different functional areas.

This study was conducted at a representative ostrich farm located in Haikou City, Hainan Province, China (19.85° N, 110.56° E). Hainan Island, the only tropical island province in China, is characterized by a warm and humid climate, abundant solar radiation, and strong air circulation—environmental conditions that are highly relevant to the generation, dispersion, and persistence of bioaerosols. Today, ostriches are distributed worldwide, and commercial farming has enabled them to adapt to a wide range of climatic conditions, including arid environments. Their natural habitats extend from semi-arid deserts to grassland regions, a characteristic that supports their successful farming in tropical areas [38]. Under such warm and humid conditions, intensive ostrich production systems may facilitate the emission and transmission of airborne microorganisms, suggesting that the investigated tropical ostrich farm provides a suitable case for bioaerosol-related investigations. The selected farm represents a mature and large-scale ostrich production system that integrates incubation, chick rearing, adult breeding, slaughtering and processing, and forage cultivation. The total farm area is approximately 4 × 10^4^ m^2^, including about 1 × 10^4^ m^2^ dedicated to adult ostrich rearing, with a regular stock of around 120 ostriches. Feeding is conducted using double-trough feeders, water is supplied through automatic drinking nozzles, and manure is manually removed every two days. To comprehensively capture the influence of different functional areas and activities on airborne microbial characteristics, 16 sampling sites were established across representative areas of the farm, as shown in Table 1 and Figure 1. Sampling was carried out during both the wet season (July 2024) and the dry season (April 2025), during which key climatic parameters, including air temperature, relative humidity, and air pressure, were recorded (Table 2) [39], to ensure data representativeness and comparability, thereby providing a robust foundation for analyzing the spatial variability of airborne microbial communities and their relationships with environmental factors.

### 2.2. Bioaerosol Sample Collection

To investigate the distribution of airborne microorganisms, sampling sites within the ostrich farm were categorized into three major areas: the breeding area, auxiliary area, and office area. At each sampling site, three samples were collected from different stages (the first, fourth, and sixth stages) of the Andersen sampler on the same day. In total, 48 samples were obtained (Table 2). The breeding areas included the brooding area, growing area, indoor ostrich rearing area, and outdoor ostrich rearing area. The supporting areas included the forage storage area, wastewater treatment area, pasture cultivation area, slaughtering area, and areas out-side the farm. The office areas comprised the product display area, kitchen, tool room, and office. The sampling strategy was informed by the spatial layout of the farm and patterns of animal activity, while also considering key factors such as the breeding cycle, animal density, spatial openness, and frequency of human intervention. This design aimed to comprehensively capture the release dynamics and spatial distribution patterns of bioaerosols under varying operational conditions. Sampling was conducted to encompass routine farm activities, including feeding, manure removal, and slaughtering, thereby ensuring the representativeness of the collected samples and enabling temporal and spatial comparisons of airborne microbial communities.

All airborne microbial samples were collected using a six-stage Andersen ZR-2002 intelligent air microbial sampler mounted on a tripod (Qingdao Zhongrui Intelligent Instrument Co., Ltd., Qingdao, China). The use of a uniform sampler model ensured consistent sampling efficiency, impactor design, and operational stability, thereby minimizing potential systematic bias arising from differences among instruments. The sampling height was set at 120 cm above the ground, which closely represents the actual exposure level of both farm personnel and animals, and aligns with conventional domestic and international bioaerosol monitoring standards (1.0–1.5 m), facilitating data comparability and reproducibility. The sampling flow rate was maintained at 28.3 L/min, with each sampling session lasting 24 h. Nutrient agar plates (70 mm in diameter) containing peptone, beef extract, sodium chloride, and agar were used in each stage of the impactor for size-fractionated sampling. After sampling, agar plates were immediately transported in a portable refrigerated box and stored at −20 °C until further analysis. To ensure data quality and prevent cross-contamination, the sampler’s interior and exterior were thoroughly disinfected with 75% ethanol before and after each sampling event. During sampling, key meteorological parameters—including daily average temperature, relative humidity, air pressure, wind speed, and wind direction were simultaneously recorded, providing essential data for subsequent analyses of factors influencing microbial distribution. To further investigate the influence of aerodynamic particle size on microbial community structure, samples from the first stage (>7.0 μm), fourth stage (2.1–3.3 μm), and sixth stage (0.65–1.1 μm) of the Andersen sampler were selected for downstream diversity analyses.

### 2.3. DNA Extraction and 16S rRNA Gene Sequencing

This study employed high-throughput 16S rRNA gene sequencing to characterize microbial community structure under different conditions and to investigate the factors influencing their composition. Total DNA was extracted from bioaerosol samples using the FastPure Soil DNA Isolation Kit (Magnetic bead) by Majorbio Bio-Pharm Technology Co. Ltd. (Shanghai, China), following the manufacturer’s instructions with minor modifications. Bacterial 16S rRNA genes targeting the V3–V4 hypervariable regions were sequenced on an Illumina MiSeq PE 300 platform (Majorbio). Sequencing data were processed and analyzed using Quantitative Insights Into Microbial Ecology (v191). High-quality sequences were clustered into operational taxonomic units (OTUs) at a 97% similarity threshold using UPARSE [40,41] (http://drive5.com/uparse/, accessed on 15 January 2026, version 7.1). Taxonomic annotation was performed against the SILVA database (v138), which provides high classification accuracy and frequent updates, enabling identification across multiple taxonomic levels including phylum, class, order, family, genus, and species.

A total of 48 bioaerosol samples were sequenced on the Illumina MiSeq high-throughput platform, generating 3,003,225 high-quality sequences. Following clustering into operational taxonomic units (OTUs) at a 97% sequence similarity threshold and subsequent taxonomic annotation, the bioaerosol samples from the ostrich farm were assigned to 24 phyla, 55 classes, 123 orders, 227 families, 484 genera, and 700 species. Rarefaction analysis indicated that species richness curves approached a plateau at a sequencing depth of 30,000 reads, with no additional taxa detected, demonstrating that the sequencing depth was sufficient to capture the majority of microbial diversity in the samples. The Shannon diversity rarefaction curves leveled off at sequencing depths exceeding 10,000 reads (see Supplementary Figure S1a), indicating that further increases in sequencing effort would provide limited additional diversity information and confirming the representativeness of the current dataset. Coverage curves approached a value of 1 across samples, further indicating that most samples were nearly fully covered, ensuring that the sequencing depth was adequate for downstream community structure analyses. Similarly, the rarefaction curves for the Simpson diversity index stabilized with increasing read numbers (see Supplementary Figure S1d), demonstrating consistent species diversity patterns among samples and reflecting the reliability of the sequencing data and the robustness of subsequent analyses [42].

### 2.4. Statistical Analysis

Alpha diversity indices, including the number of observed species, Chao1 richness estimator, Shannon index, and Simpson index, were calculated using Mothur software (v1.30.2). Statistical comparisons of alpha diversity among different groups were performed in R Language (v3.3.1) using the stats package with the Kruskal–Wallis test. The Chao1 index was employed to estimate species richness, the Shannon index to assess community diversity, and the Simpson index to evaluate species evenness within communities. Beta diversity was analyzed based on Bray–Curtis distance matrices, and principal coordinates analysis (PCoA) was applied to assess differences in community structure. Differential taxa among functional areas were identified using LEfSe (Linear Discriminant Analysis Effect Size) [43] (LDA score > 2, *p* < 0.05).

## 3. Results

### 3.1. Bacterial Community Diversity Analysis

**Figure 2:**
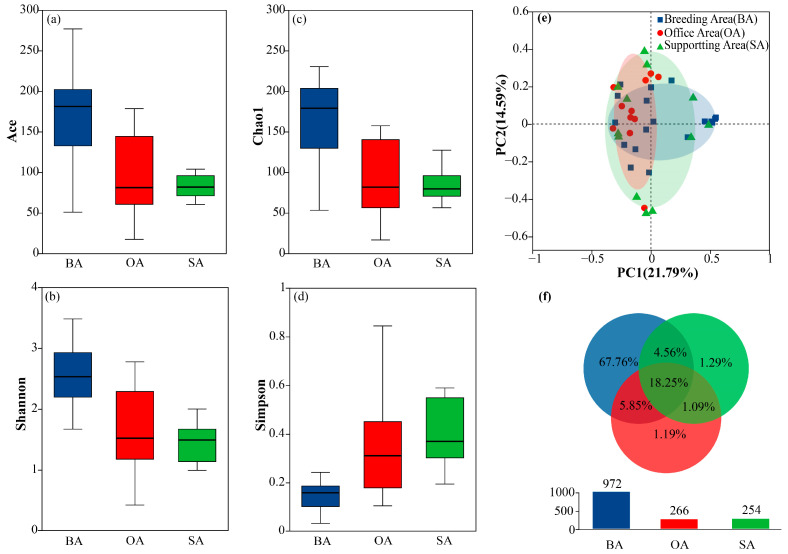
Boxplots of alpha diversity indices of air samples from the ostrich breeding farm: ACE index (**a**), Shannon index (**b**), Chao1 index (**c**), and Simpson index (**d**). Two-dimensional ordination plot based on PCoA analysis (**e**). Venn diagram showing OTUs among the three functional area groups (**f**); each ellipse represents one group, and overlapping areas indicate shared OTUs. Numbers within each segment denote the number of OTUs.

To evaluate within-sample species diversity, α-diversity analyses were conducted, with higher Ace and Chao1 indices indicating greater species richness, a higher Shannon index reflecting increased diversity, and a lower Simpson index suggesting a more even species distribution. Samples from the breeding area exhibited the highest diversity, with Ace (Figure 2a), Shannon (Figure 2b), and Chao1 (Figure 2c) indices significantly higher than those from the supporting and office areas (*p* < 0.05).

β-diversity analyses further revealed differences in community composition between samples. PCoA based on Bray–Curtis distances (Figure 2e) showed distinct clustering of samples from the breeding, supporting, and office areas, indicating substantial heterogeneity in bacterial community structure. Additionally, OTU counts were used to evaluate sample diversity and richness [44], and Venn diagrams were generated using jvenn (Figure 2f) [45]. The results indicated that 184 OTUs were shared among the three areas, accounting for 18.25% of the total. The breeding area contained 683 unique OTUs, whereas the supporting and office areas contained 89 and 106 unique OTUs, respectively. Further bar chart analysis showed that the total number of OTUs in the breeding area (972) was substantially higher than in the supporting (254) and office areas (266), consistent with the α-diversity results.

**Figure 3:**
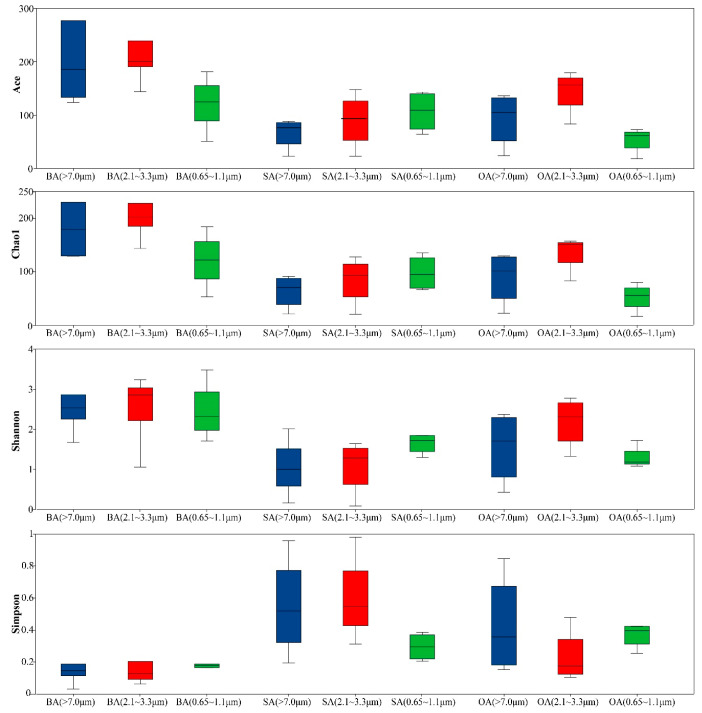
Diversity analysis of airborne microbial communities across different particle size fractions.

Building on the overall diversity patterns, α-diversity analyses were conducted to further explore the influence of airborne particle size on microbial community distribution in three particle size fractions (Figure 3). The results showed that Ace and Chao1 indices decreased with decreasing particle size, indicating a reduction in microbial species richness from large to small particles. In contrast, the Shannon index reached its maximum and the Simpson index its minimum in the medium particle size fraction (2.1–3.3 μm), reflecting the most even and complex community structure at this size. Significant differences were also observed among particle size fractions across different functional areas. The breeding area exhibited significantly higher Ace and Chao1 indices than the supporting and office areas across all particle size fractions (*p* < 0.05).

### 3.2. Analysis of Airborne Microbial Composition

**Figure 4:**
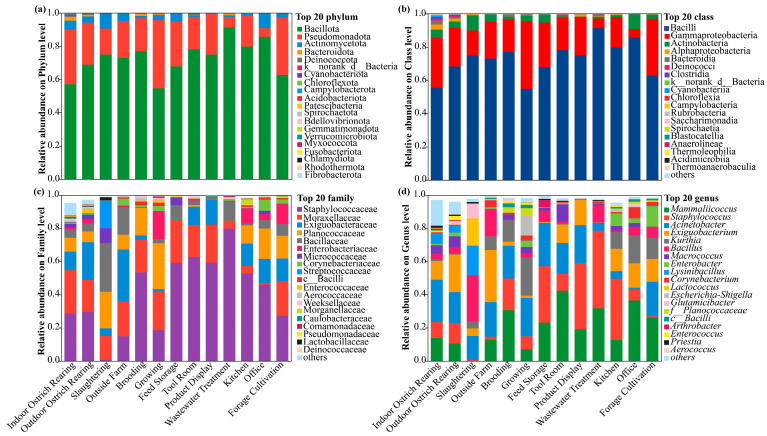
Composition of airborne microbial communities in the ostrich farm. Relative abundances at the phylum (**a**), class (**b**), family (**c**), and genus (**d**) levels.

To further elucidate the compositional differences in airborne microbial communities across functional areas, the samples from the breeding, supporting, and office areas of the ostrich farm were analyzed at the phylum, class, family, and genus levels (Figure 4). Taxa with relative abundances below 1% in all samples were grouped into the “Others” category. At the phylum level, Bacillota, Proteobacteria, and Actinobacteriota were the dominant phyla, collectively accounting for over 90% of the community. Bacillota showed significantly higher relative abundance in the breeding area (65–85%, *p* < 0.05). At the class level, Bacilli and γ-Proteobacteria were predominant. At the family and genus levels, Staphylococcaceae, Bacillaceae, and Moraxellaceae were dominant, corresponding to the genera *Staphylococcus*, *Bacillus*, and *Acinetobacter*.

**Figure 5:**
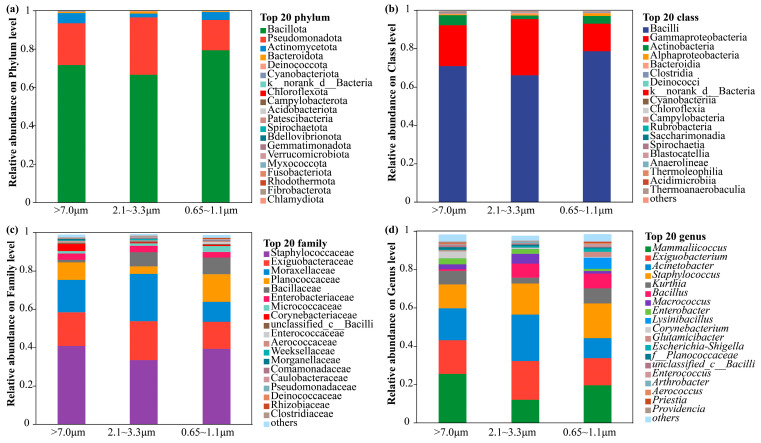
Stacked bar plots of the relative abundances of airborne microbial communities across different particle size fractions at the phylum (**a**), class (**b**), family (**c**), and genus (**d**) levels.

We further examined the compositional differences in bacterial communities across three particle size fractions (Figure 5). At the phylum level, Bacillota was the dominant group across all particle size fractions, exhibiting the highest relative abundance in the large particle fraction. Proteobacteria showed increased relative abundance in the medium-sized fraction, whereas Actinobacteriota and Bacteroidota were enriched in the small particle fraction. At the genus level, Staphylococcus, Bacillus, and Acinetobacter were identified as the core genera. *Staphylococcus* and *Acinetobacter* reached their highest relative abundances in the medium-sized fraction (2.1–3.3 μm). The small particle fraction showed higher relative abundances of genera such as *Bacillus* and *Lactobacillus*.

**Figure 6:**
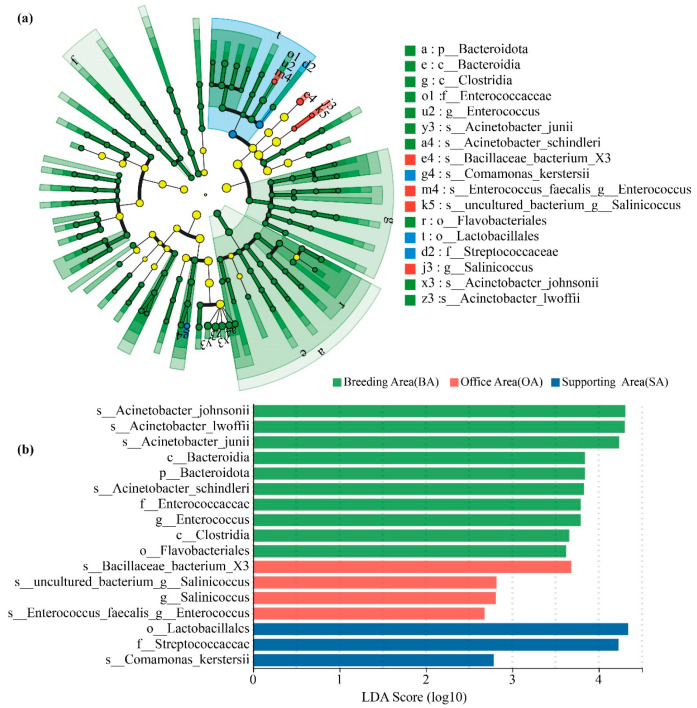
Microbial community analysis of ostrich farm samples using LEfSe. Taxonomic cladogram illustrating the hierarchical relationships of major taxa from phylum to species (from inner to outer rings) across the breeding, office, and supporting areas (**a**). Node colors and sizes represent microbial taxa with significant differences and varying relative abundances. LEfSe results for the three functional areas (**b**).

Differential analysis of bacterial taxa among functional areas was further conducted using LEfSe (Linear Discriminant Analysis Effect Size) with an LDA score cutoff >4 and *p* < 0.05 (Figure 6a). The results revealed that the breeding area was significantly enriched with genera such as *Staphylococcus*, *Acinetobacter*, and *Micrococcus*, whereas the office area was enriched in taxa from the phylum Actinobacteriota. Several characteristic genera associated with feed or surface contact were detected in the supporting area. Figure 6b illustrates the phylogenetic distribution of these differential taxa from phylum to species level across the three functional areas, providing insights into the ecological characteristics and functional differentiation of airborne bacterial communities in these distinct areas.

### 3.3. Relative Abundance of Airborne Samples

**Figure 7:**
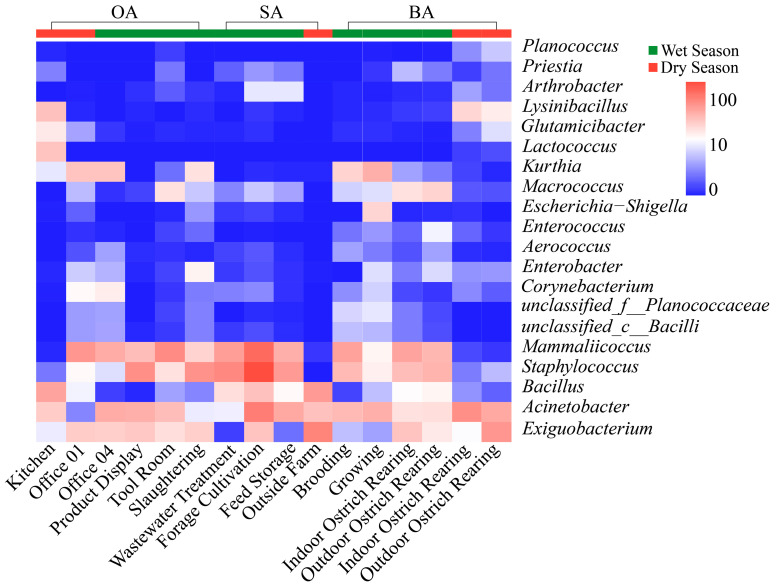
Heatmap of the relative abundances of the 20 most dominant bacterial genera in the ostrich farm.

A heatmap was generated using the log-transformed abundances of the top 20 OTUs (Figure 7), revealing significant differences in community structures among sampling points and seasons, with dominant taxa exhibiting discernible spatiotemporal patterns. *Bacillus*, *Staphylococcus*, *Acinetobacter*, and *Brevibacillus* were consistently abundant across most samples, constituting the core microbiota of airborne bioaerosols in the study area. Seasonal variation was observed, with *Bacillus* and *Staphylococcus* showing higher relative abundances in the dry season, whereas *Enterococcus* and *Bacillus subtilis*-related taxa were enriched in the wet season. Cluster analysis further demonstrated distinct genus-level groupings among functional areas, with breeding and related functional areas (e.g., brooding area) exhibiting similar bacterial community structures, while office areas and external farm environments differed markedly from these animal-occupied areas.

### 3.4. Detectable Metagenomic Antibiotic Resistance

**Figure 8:**
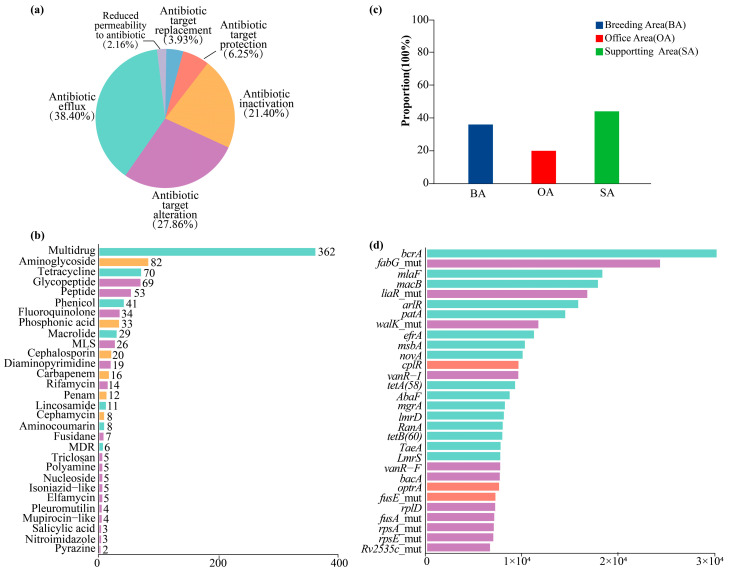
Antibiotic resistance in airborne samples. Proportions of different antibiotic resistance mechanisms (**a**); Relative abundances of ARGs to the antibiotic classes. Numbers on the bars represents detected ARG numbers of each antibiotic class (**b**); Proportions of airborne ARGs across different functional areas of the ostrich farm (**c**); Relative abundances of the top 30 ARGs(RPKM) (**d**).

Metagenomic analysis of airborne samples from the ostrich farm identified a total of 638 potential ARGs, including seven classified as high-risk genes (*dfrA1*, *dfrA12*, *dfrA14*, *tetM*, *mcr1*, *ermB*, and *sul1*) [46]. Among the resistance mechanisms, efflux pumps accounted for the largest proportion (approximately 38%), followed by target alteration (approximately 28%), drug inactivation (approximately 21%) (Figure 8a). These ARGs are associated with 34 antibiotic classes (Figure 8b), among which resistance genes conferring resistance to multidrug (362 genes), aminoglycoside (82 genes), tetracycline (70 genes), and glycopeptide (69 genes) were dominant. The distribution of ARGs across functional areas revealed that the supporting areas of the ostrich farm exhibited the highest detection rates, followed by the breeding areas, whereas the office areas showed comparatively lower levels (Figure 8c). Further analysis of the relative abundance of the top 30 ARGs indicated that efflux pump-associated genes, including *bcrA*, *fabG_mut*, *mlaF*, *macB*, *liaR_mut*, *arlR*, *patA*, and *walK_mut*, were the most abundant (Figure 8d).

β-Lactam resistance genes were detected in the bioaerosol samples, including classical ESBL-associated genes such as *blaTEM-1* and *blaSHV-61*, suggesting potential public health relevance. In addition, several *CTX-M* variants were also identified, including *blaCTX-M-140*, *blaCTX-M-14*, *blaCTX-M-55*, and *blaCTX-M-60*, although their overall abundance was extremely low. Unlike the globally predominant clinical *CTX-M* lineages, the detected *CTX-M* variants belonged to relatively rare subtypes, which may partially explain their limited representation in the airborne resistome. In contrast, OXA-type β-lactamase genes dominated the β-lactam resistance profile, a pattern consistent with previous observations in environmental and livestock-associated bioaerosols but distinct from those typically reported in hospital settings.

In addition to β-lactam resistance, fluoroquinolone resistance determinants were widely detected in the airborne samples. Multiple chromosomal target alteration-associated genes, including *gyrA*, *gyrB*, and *parC*, were broadly distributed, with particularly high abundances observed for *Staphylococcus aureus gyrB*, *S. aureus gyrA*, and *S. aureus parC*, followed by *Acinetobacter baumannii gyrA* and *parC*. Unlike ESBL genes that are commonly acquired through horizontal gene transfer, resistance mediated by *gyrA*, *gyrB*, and *parC* generally reflects long-term antibiotic selection pressure. From a One Health perspective, the detection of these clinically important fluoroquinolone resistance determinants in bioaerosols is of considerable concern, given that fluoroquinolones are critically important antimicrobials in human medicine.

### 3.5. Analysis of Carbon Cycle-Related Functional Genes

To investigate the enzymatic repertoire involved in complex polysaccharide degradation, we further analyzed the genes encoding CAZymes within the microbial communities of ostrich farm air samples [47]. Functional annotation of CAZyme modules containing signal peptides was performed using the CAZy database, and the resulting sequences were classified into six major categories associated with carbohydrate synthesis, degradation, and recognition. These categories included glycosyltransferases (GTs), glycoside hydrolases (GHs), carbohydrate esterases (CEs), carbohydrate-binding modules (CBMs), polysaccharide lyases (PLs), and auxiliary activity enzymes (AAs) [48].

**Figure 9:**
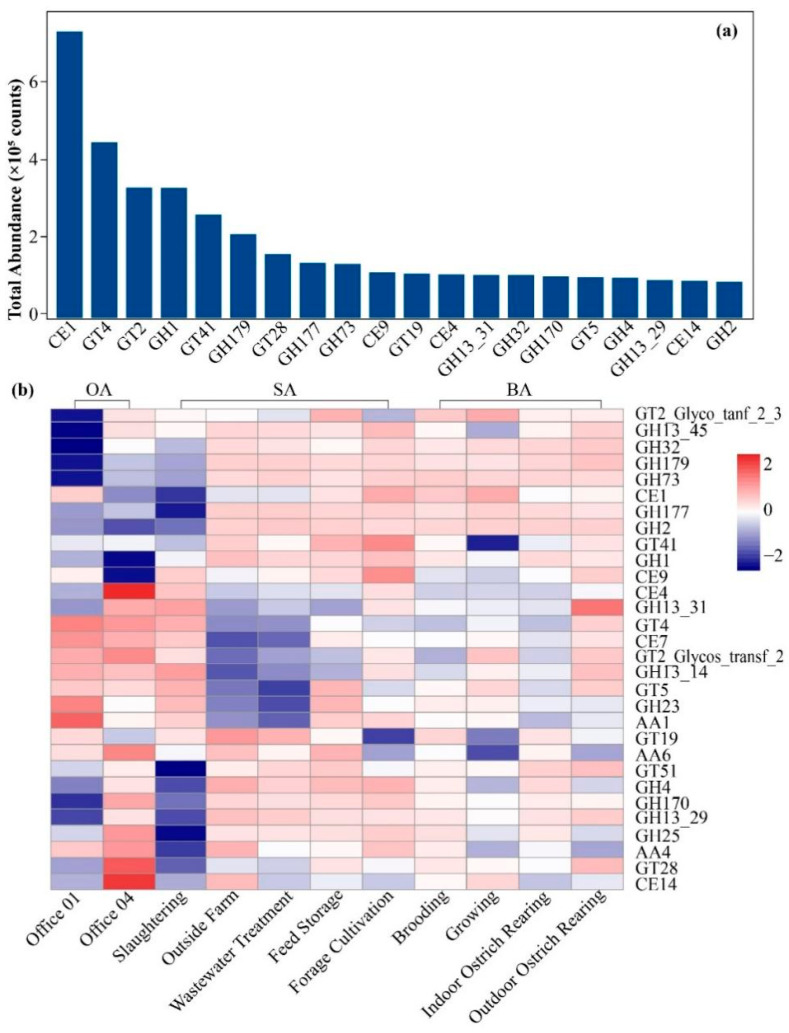
Bar plot showing the top 20 most abundant CAZy families in airborne samples from the ostrich farm (**a**). Heatmap illustrating the distribution of CAZyme module abundances in airborne samples (log_10_-transformed values) (**b**).

Among all annotated CAZyme modules, 35 CAZyme families accounted for more than 1% of the total annotations. As shown in Figure 9a, *CE1*, *GT4*, and *GT2_Glycos_transf_2* were the dominant families, exhibiting significantly higher abundances than the others. In addition, families such as *GH1*, *GH41*, *GH79*, *GT28*, and *GH177* also displayed relatively high abundances, collectively constituting the major components of carbohydrate-active enzymes in the air samples. In contrast, families such as *GH13_14*, *AA1*, and *GH23* were detected at relatively low abundances.

Cluster analysis of CAZyme family abundances at the genus level revealed distinct clustering patterns among samples from different functional areas (Figure 9b). The CAZyme family profiles in the breeding areas and their associated functional areas were relatively similar, whereas samples from the office area and the external farm environment exhibited markedly different distributions. Further analysis revealed that families such as *GT2_Glyco_transf_2_3*, *GH73*, and *CE1* were relatively abundant across most supporting areas, while *GH13_14*, *AA1*, and *GH23* exhibited more localized distributions.

### 3.6. Analysis of Microbial Nitrogen Functional Genes

**Figure 10:**
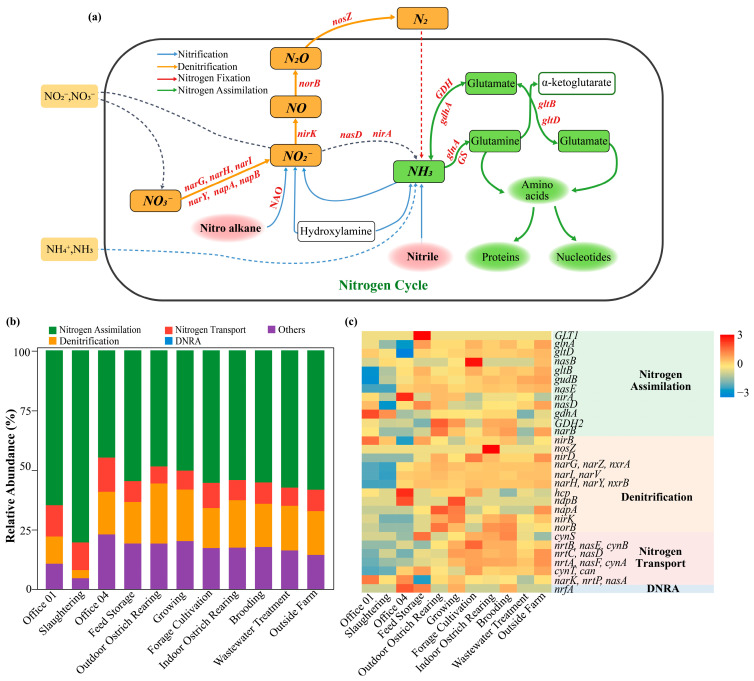
Schematic representation of major nitrogen metabolism pathways and key enzymes (KEGG KOs) (**a**). Blue arrows represent nitrification, orange arrows represent denitrification, red arrows rep-resent nitrogen fixation, and green arrows represent nitrogen assimilation. Relative abundances of nitrogen cycle functional genes across samples (**b**). Heatmap of nitrogen metabolism-related functional genes (**c**).

Based on metagenomic annotation, a functional map of nitrogen cycling within the airborne microbial community was constructed (Figure 10). As shown in Figure 10a, the annotated functional genes were primarily associated with nitrogen assimilation, denitrification, nitrate/nitrite transport, and dissimilatory nitrate reduction to ammonium (DNRA), whereas canonical nitrogen fixation (e.g., *nif* genes) and nitrification (e.g., *amoA*, *hao*) genes were not detected. Nitrogen assimilation-related genes such as *glnA*, *gltB/D*, *gdhA*, and *gudB* were the most abundant across most samples (Figure 10b), accounting for approximately 60–80% of the total nitrogen metabolism genes. Denitrification-related genes (*narGHIJ*, *napAB*, *nirK/S*, *norB*, *nosZ*) were detected at relatively lower proportions (approximately 5–20%) but showed higher relative abundances in the breeding and wastewater treatment areas. DNRA-related genes (e.g., *nrfA*) were detected in some samples. In addition, nitrate/nitrite transporter genes (e.g., n*rtA–C*, *nasA–E*, *cynA–B*) were widely distributed across all samples. The heatmap of nitrogen metabolism genes (Figure 10c) revealed clear spatial heterogeneity. Denitrification-related genes (*narG*, *nirK*, *norB*, *nosZ*) were enriched in the breeding and wastewater treatment areas, whereas nitrogen assimilation genes (*glnA*, *gltB/D*, *gdhA*) were consistently abundant across all functional areas.

## 4. Discussion

The significantly higher α-diversity indices observed in the breeding area indicate that this functional zone harbors more complex and diverse airborne microbial communities than the supporting and office areas. This pattern is consistent with previous studies in poultry and livestock farming environments [49], in which intensive animal activity, fecal deposition, and feed residues enhance microbial inputs into the air. The elevated diversity likely reflects continuous resuspension of microbe-laden particles driven by animal movement and routine management practices. β-diversity analysis further revealed pronounced spatial heterogeneity in bacterial community composition among different functional areas, consistent with observations from swine farm dust samples [50], highlighting the strong influence of functional zoning on airborne microbial assemblages. Particle size exerted a strong influence on microbial diversity distribution. Larger particles carried higher species richness, likely due to their capacity to transport aggregates of microorganisms attached to organic debris. In contrast, the medium-sized fraction (2.1–3.3 μm) exhibited the highest community evenness and complexity, as indicated by Shannon and Simpson indices, suggesting a balance between suspension efficiency and microbial survival. Similar particle size-dependent patterns have been widely reported in bioaerosol studies [51]. The presence of a shared core microbiome across functional areas further suggests that airflow and human activity promote microbial dispersion and partial homogenization [52].

Across all functional areas, Bacillota, Proteobacteria, and Actinobacteriota dominated the airborne microbial communities, consistent with previous reports from poultry farm aerosols [53]. The enrichment of Bacillota in the breeding area supports its role as a core component of bioaerosols in animal housing environments, reflecting its resilience to environmental stress and association with animal-derived substrates. At the genus level, the prevalence of *Staphylococcus*, *Bacillus*, and *Acinetobacter* underscores their strong adaptability to airborne transmission and environmental stress. Differences among particle size fractions further emphasize the role of aerodynamic properties in structuring airborne bacterial assemblages. The enrichment of *Staphylococcus* and *Acinetobacter* in medium-sized particles may be linked to enhanced suspension capacity and prolonged atmospheric residence time, whereas environmentally resilient taxa detected in smaller particles likely reflect selective survival under harsher conditions such as desiccation and ultraviolet radiation stress. Seasonal variation in dominant taxa, together with the similarity among breeding-related functional areas compared with the more distinct profiles of office and external environments, suggests that both temporal factors and the intensity of human–animal activities jointly shape airborne microbial community composition [54].

The detection of clinically relevant ARGs indicates the presence of an airborne resistome with potential ecological and health relevance at the genetic level, rather than direct evidence of phenotypic resistance or active dissemination. The predominance of multidrug, aminoglycoside, tetracycline, and glycopeptide resistance genes suggests that ostrich farms may function as hotspots for the circulation of clinically relevant ARGs. Previous studies have identified Lactobacillus, Staphylococcus, and Acinetobacter as major ARG carriers in poultry manure and farm aerosols [55,56], with Staphylococcus aureus frequently detected in farm ai [57]. Due to its pathogenicity and broad host range, *S. aureus* represents a key biosafety concern [58], while *Acinetobacter* and its associated phages are recognized as important drivers of ARG dissemination and microbial evolution [59]. The higher ARG abundance observed in supporting areas suggests that these zones may serve as secondary dissemination hotspots, whereas breeding areas likely represent primary ARG sources and office areas pose comparatively lower risks. The widespread detection of aminoglycoside resistance genes aligns with previous findings from pig and poultry farms [60,61], as well as urban air environments [62]. However, a global survey of air samples from 19 cities revealed that the overall abundance of *aac*(6′)-Ib and *aac*(6′)-II was considerably lower than that of quinolone- and β-lactam-resistance genes [63]. Taken together, these results indicate that ostrich farms may function as potential reservoirs of airborne ARGs, emphasizing the need for targeted monitoring and further functional validation.

The dominance of CAZyme families such as CE1, GT4, and GT2_Glycos_transf_2 indicates that carbohydrate degradation and transformation functions are prominent within the airborne microbial communities of the ostrich farm. These families are primarily associated with the metabolism of cellulose, hemicellulose, and related polysaccharides [64], reflecting the availability of organic substrates derived from feed residues, feces, skin debris, and bedding materials [33,65]. The enrichment of glycoside hydrolase and glycosyltransferase families is consistent with the metabolic demands of heterotrophic microbial communities dominated by Bacillota and Actinobacteriota. In contrast, low-abundance CAZyme families likely contribute to niche-specific adaptive functions but play a limited role in overall carbon turnover.

Nitrogen cycling is a complex biogeochemical process involving multiple redox transformations from oxidized nitrate (NO_3_^−^, +5) to reduced ammonia (NH_3_, −3), mediated by diverse microbial taxa [66]. The dominance of nitrogen assimilation-related genes indicates that nitrogen metabolism within the airborne microbial community is primarily governed by assimilatory pathways, in which inorganic nitrogen species such as NO_3_^−^ and NH_4_^+^ are incorporated into organic nitrogen compounds (e.g., glutamate and glutamine) to support microbial growth and metabolism. The enrichment of denitrification-related genes in the breeding and wastewater treatment areas may be associated with localized environmental conditions, including elevated organic load or reduced oxygen availability, which favor reductive nitrogen transformation processes. In addition, the presence of DNRA-related genes suggests the potential for nitrate respiration leading to ammonium production, thereby enhancing nitrogen retention within the airborne microbial community. The widespread occurrence of nitrate and nitrite transporter genes further reflects a strong capacity for inorganic nitrogen uptake and transmembrane transport.

Several limitations should be acknowledged. First, the 24 h Andersen air sampling protocol may introduce bias related to microbial viability and activity, potentially leading to the underrepresentation of sensitive taxa; this potential limitation is unlikely to substantially affect the overall community patterns or comparative conclusions. Second, sampling locations were not fully identical between dry and rainy seasons, and non-breeding areas were defined based on the presence of human activity without quantitative assessment of activity intensity, which may introduce spatial heterogeneity. In addition, extreme weather events during the dry season prevented sampling at certain sites, resulting in a reduced sample size. These factors may increase uncertainty in seasonal comparisons and should be addressed in future studies through refined site matching and quantitative evaluation of human activity intensity. This study is based on DNA-level metagenomic analysis. Therefore, the detected ARGs represent genetic potential rather than confirmed expression, phenotypic resistance, or horizontal gene transfer. Future studies integrating metatranscriptomics, cultivation-based assays, and host association analysis are needed to further assess functional activity and transmission risk.

This study demonstrates that bioaerosols in ostrich farms harbor diverse microbial communities with pronounced spatial and seasonal heterogeneity, elevated abundances of antimicrobial resistance genes, and functional potential related to carbon and nitrogen cycling. By integrating microbial community analysis with resistome and functional profiling, these findings provide new insights into the ecological roles and biosafety risks of bioaerosols in specialty livestock systems and offer a scientific basis for targeted management and risk mitigation strategies.

## 5. Conclusions

This study employed 16S rRNA gene sequencing combined with metagenomic analysis of multi-particle-size air samples to elucidate the diversity patterns, ARG profiles, and carbon and nitrogen cycling potential of microbial communities in a tropical ostrich farm. Airborne bacterial communities were dominated by Bacillota, Proteobacteria, and Actinomycetota, with *Staphylococcus*, *Bacillus*, and *Acinetobacter* identified as the predominant genera. Analysis across particle size fractions revealed that larger particles (>7.0 μm) harbored the highest microbial richness, whereas medium-sized particles (2.1–3.3 μm) exhibited the greatest diversity; small particles were enriched with environmentally resilient taxa such as *Brevibacillus* and *Corynebacterium*, indicating that aerodynamic properties strongly influence microbial adhesion and dispersal. Comparisons among functional areas showed that breeding area samples exhibited the highest microbial diversity and ARG abundance, likely reflecting the influence of animal activity, fecal dust, and feed-derived bioaerosols. Metagenomic profiling revealed a diverse airborne resistome in the ostrich farming environment. These findings indicate a genetic reservoir of antibiotic resistance determinants, rather than direct evidence of phenotypic resistance or active transmission, underscoring the need for continued surveillance and functional validation in specialty livestock systems. Carbon metabolism-related genes were primarily associated with glycoside hydrolases and glycosyltransferases, demonstrating a strong capacity of airborne microbial communities for organic matter degradation and energy acquisition. Nitrogen cycling genes were mainly involved in airborne dissemination in nitrogen assimilation (*nasA–E*, *glnA*, *gltB/D*, *gdhA*) and denitrification (*narG*, *nirK*, *norB*, *nosZ*), with DNRA-related genes (*nrfA*) also detected; however, genes associated with nitrogen fixation (*nif*) or nitrification (*amoA*, *hao*) were absent, indicating that airborne microbes primarily participate in inorganic nitrogen uptake, reduction, and organic incorporation rather than nitrogen fixation or oxidation. Denitrification genes were particularly enriched in the breeding area, suggesting that local hypoxic conditions may enhance the potential for N_2_O and other nitrogen oxide emissions.

The results indicate that airborne microbial communities exhibit pronounced differences in diversity and metabolic function across particle size fractions and functional areas. Future studies should integrate multi-seasonal and multi-site monitoring with transcriptomic and metabolomic approaches to further elucidate the ecological functional activity of airborne microbiota and the mechanisms underlying ARG dissemination. These insights may provide reference information for managing air quality and public health risks under the specific conditions of the investigated tropical ostrich farm.

## Figures and Tables

**Table 1:** Distribution of sampling sites at the ostrich farm.

Functional Area	Sampling Site	Number of Sampling Sites	Samples Per Site
Breeding Area(BA)	Brooding	1	3
	Growing	1	3
	Indoor Ostrich Rearing	2	6
	Outdoor Ostrich Rearing	2	6
Supporting Area(SA)	Feed Storage	1	3
	Wastewater Treatment	1	3
	Forage Cultivation	1	3
	Slaughtering	2	6
	Outside Farm	1	3
Office Area(OA)	Product Display	1	3
	Kitchen	1	3
	Tool Room	1	3
	Office	1	3
Total Number of Samples		16	48

**Table 2:** Daily average meteorological parameters at the ostrich farm.

Time	Sampling Site	Weather	Air Pressure (kPa)	T (°C)	RH (%)
23 July 2024	Brooding	Wet	99.35	39.2	53.7
23 July 2024	Growing	Wet	99.47	37.8	54.3
24 July 2024	Indoor Ostrich Rearing	Wet	99.25	36.3	58.2
24 July 2024	Outdoor Ostrich Rearing	Wet	99.25	36.3	58.2
25 July 2024	Feed Storage	Wet	99.20	37.4	64.7
25 July 2024	Tool Room	Wet	99.30	36.5	64.0
27 July 2024	Product Display	Wet	99.85	34.5	70.0
27 July 2024	Wastewater Treatment	Wet	100.04	35.5	60.3
29 July 2024	Kitchen	Wet	99.97	34.2	69.9
29 July 2024	Office	Wet	100.05	33.0	75.4
30 July 2024	Forage Cultivation	Wet	100.01	39.1	51.6
30 July 2024	Outside Farm	Wet	100.21	39.4	51.6
3 April 2025	Indoor Ostrich Rearing	Dry	99.97	34.2	69.9
4 April 2025	Outdoor Ostrich Rearing	Dry	100.05	33.0	75.4
5 April 2025	Slaughtering	Dry	100.21	39.4	51.6
5 April 2025	Outside Farm	Dry	100.22	39.3	51.6

## Data Availability

The data presented in this study are available upon request from the corresponding author. The raw sequencing data were generated from a commercial ostrich farm; therefore, their ownership and access are subject to the farm operator, but they can be made available upon reasonable request. but can be provided upon reasonable request.

## Supplementary Materials

The following supporting information can be downloaded at: https://www.mdpi.com/article/10.3390/ani16060880/s1, Figure S1: Rarefaction curves of bacterial communities based on different diversity indices.
