# Supplementary material for: Airborne Microbiome of Tropical Ostrich Farms: Diversity, Antibiotic Resistance, and Biogeochemical Cycling Potential
Source: Animals (Basel). 2026 Mar 12;16(6):880. doi: 10.3390/ani16060880 (PMC13023248; doi:10.3390/ani16060880)
Supplement: Supplementary file 1 [file animals-16-00880-s001.zip › animals-4079997-supplementary.pdf]

Airborne Microbiome of Tropical Ostrich Farms: Diversity, Antibiotic Resistance, and Biogeochemical Cycling Potential

Yu Yang, Junchi Wang, Zetong Wang, Cheng Li *, Xiaolei Hu, Songdi Liao and Lizhi Wang

Key Laboratory of Agro-Forestry Environmental Processes and Ecological Regulation of Hainan Province, School of Environmental Science and Engineering, Hainan University, Haikou 570228, China; yangyu_mail@163.com (Y.Y.); wangjunchi987@163.com (J.W.); 24210830000017@hainanu.edu.cn (Z.W.); 996173@hainanu.edu.cn (X.H.); 996983@hainanu.edu.cn (S.L.); wanglz@hainanu.edu.cn (L.W.)

* Correspondence: 996154@hainanu.edu.cn; Tel.: +86-180-117-008-76

Supplementary material

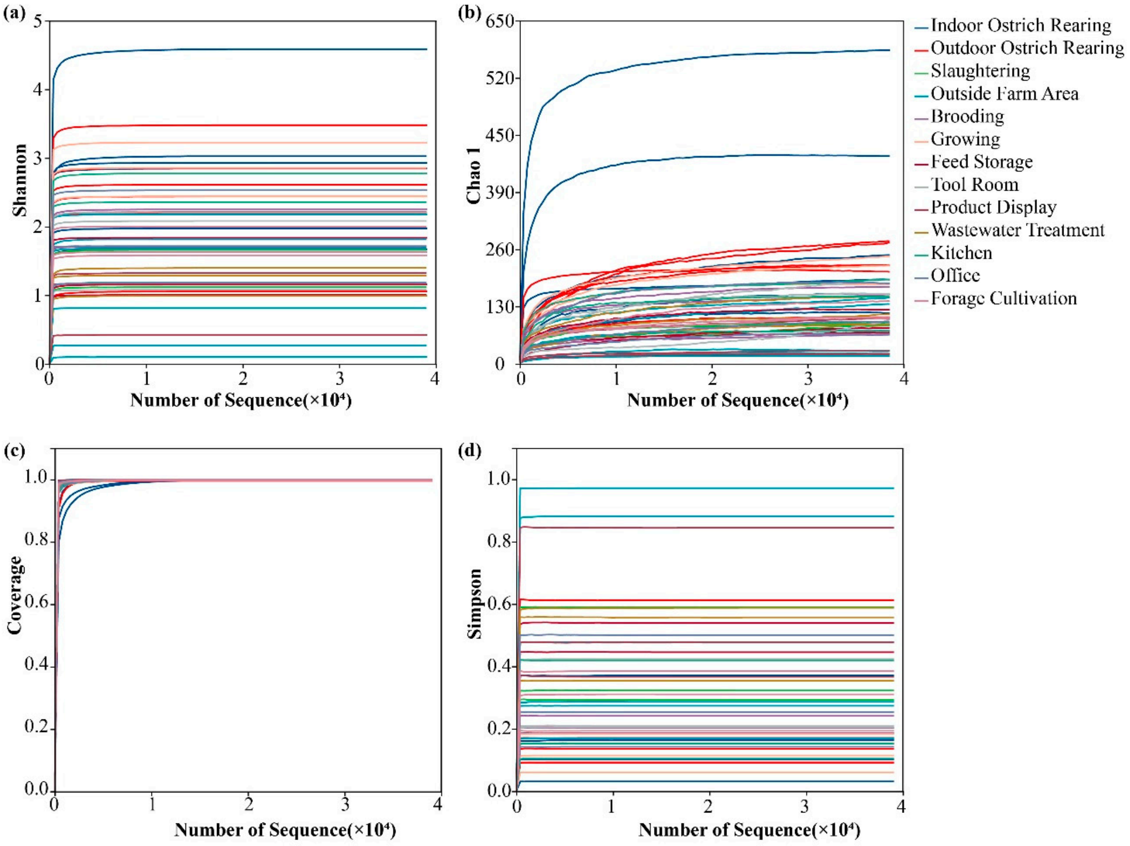

Figure S1. Rarefaction curves of bacterial communities based on different diversity indices. Rarefaction curves based on the Shannon index (a), Chao1 index (b), Shannon index (c), and Simpson index (d). The curves tend to approach saturation, indicating that the sequencing depth was sufficient to capture the majority of bacterial diversity within the samples..
